# Biotechnological Aspects and Mathematical Modeling of the Biodegradation of Plastics under Controlled Conditions

**DOI:** 10.3390/polym14030375

**Published:** 2022-01-18

**Authors:** Yvan Baldera-Moreno, Valentina Pino, Amelia Farres, Aparna Banerjee, Felipe Gordillo, Rodrigo Andler

**Affiliations:** 1 Facultad de Ciencias Básicas, Universidad Católica del Maule, Talca 3460000, Chile; yvan.baldera@alu.ucm.cl; 2 Escuela de Ingeniería en Biotecnología, Centro de Biotecnología de los Recursos Naturales (Cenbio), Universidad Católica del Maule, Talca 3460000, Chile; valentina.pino@alu.ucm.cl (V.P.); fgordillo@ucm.cl (F.G.); 3 Departamento de Alimentos y Biotecnología, Universidad Nacional Autónoma de México, Ciudad de Mexico 04510, Mexico; amelia.farres@gmail.com; 4 Centro de Investigación de Estudios Avanzados del Maule, Vicerrectoría de Investigación y Postgrado, Universidad Católica del Maule, Talca 3460000, Chile; abanerjee@ucm.cl

**Keywords:** microbial degradation, plastic biodegradation, plastic pollution, polymer degradation, modeling

## Abstract

The strong environmental impact caused by plastic pollution has led research to address studies from different perspectives. The mathematical modeling of the biodegradation kinetics of solid materials is a major challenge since there are many influential variables in the process and there is interdependence of microorganisms with internal and external factors. In addition, as solid substrates that are highly hydrophobic, mass transfer limitations condition degradation rates. Some mathematical models have been postulated in order to understand the biodegradation of plastics in natural environments such as oceans. However, if tangible and optimizable solutions are to be found, it is necessary to study the biodegradation process under controlled conditions, such as using bioreactors and composting systems. This review summarizes the biochemical fundamentals of the main plastics (both petrochemical and biological origins) involved in biodegradation processes and combines them with the main mathematical equations and models proposed to date. The different biodegradation studies of plastics under controlled conditions are addressed, analyzing the influencing factors, assumptions, model developments, and correlations with laboratory-scale results. It is hoped that this review will provide a comprehensive overview of the process and will serve as a reference for future studies, combining practical experimental work and bioprocess modeling systems.

## 1. Introduction

Plastics were developed as highly resistant materials, which resulted in a high number of applications in diverse industrial sectors, such as food, medical devices, construction, and automotive. Unfortunately, the current level of use has resulted in a very serious menace to life in the oceans and in terrestrial ecosystems, and some of the original disposal proposals, such as landfills and incineration processes, are highly disruptive to the environment [1].

Plastics are categorized according to their chemical building blocks and their manufacturing processes. In addition, plastics contain several additives such as plasticizers or flame retardants. Therefore, plastic degradation is not a general process; it must be designed for each specific plastic or mixture, especially if there is a desire to recover the building blocks for use in other chemical processes, as the idea of the circular economy postulates. Plastic degrades in the environment through the action of oxygen, weathering, mechanical factors, temperature, and microbial colonization in a sequence of events that may take hundreds of years [2]. The mechanisms designed to destroy plastics must take into account the energy put into building the polymer structure, which leads to the use of high temperatures, high pressures, and special organic or inorganic catalysts for the development of thermochemical processes [3,4].

**Figure 1:**
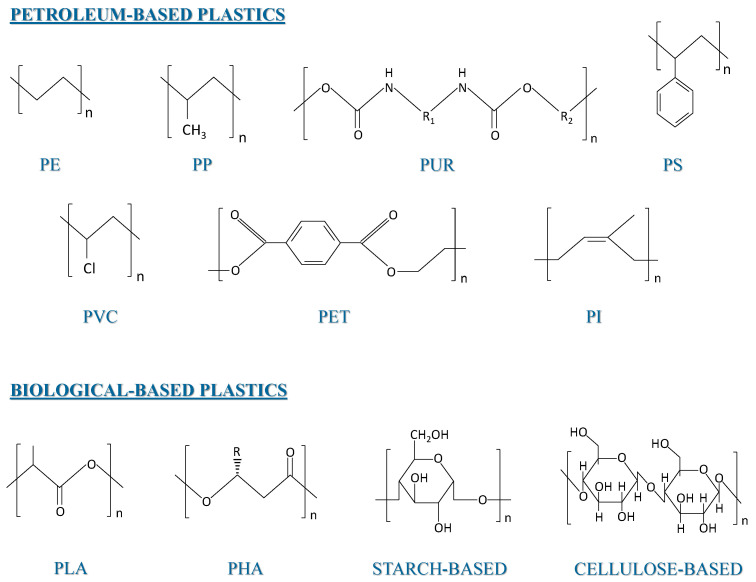
Chemical structure of petroleum-based and biological-based plastics.

A promising strategy is based on the use of microorganisms that degrade these materials when thrown in landfills or composting sites. Some very effective strains have been isolated, which are able to degrade different polymers [5]. Microorganisms use metabolic pathways where enzymes are the central catalysts; therefore, the use of enzymes to degrade polymers has been explored as a sustainable bioremediation process [6]. Synthetic petroleum-derived polymers are the target for the development of new environmentally friendly/eco-friendly biotransformation processes, with polyethylene (PE), polyethylene terephthalate (PET), poly vinyl chloride (PVC), and polypropylene (PP) being the most common polymers and the bulk of most commodity plastics. One of the difficulties in the biodegradation (BD) of plastics is the high prevalence of C–C bonds, especially for PP, PE, polystyrene (PS), and PVC, while polyurethane (PU) has a urethane bond and PET an ester bond, which are both hydrolysable [7] (Figure 1).

For bio-based plastics, such as polylactic acid (PLA) and polyhydroxyalkanoates (PHAs), as well as starch-based and cellulose-based plastics, biodegradation occurs at higher rates, since the corresponding glycosidic and ester bonds are easily catalyzed by microorganisms, thus making polymer fragmentation much faster. In addition, the necessary enzymes for the uptake of monomers coming from bio-based plastics are, in general, highly available in microorganisms.

An ideal solution to plastic accumulation would lie in the design of environmentally friendly/eco-friendly recycling processes that lead to the use of chemicals generated through them or to energy generation, without an increase in pollution. In this scenario, composting systems are considered some of the most promising alternatives, due to the possibility of controlling important parameters, such as humidity, pH, aeration, and temperature [8,9,10]. In order to understand and optimize composting systems, simulated composting systems are often utilized [11].

Several studies have focused on finding mathematical models to understand the biodegradation rates in marine environments [12,13,14]. However, it is of the utmost urgency to establish models that allow researchers to find out the impact of each of the factors involved during biodegradation, such as temperature, humidity, and aeration in controlled environments, facilitating the understanding, optimization, and application of strategies with respect to these factors and their interactions, which is key to developing highly efficient biodegradation processes. There are only a few studies in the literature that allow a deep and systematic understanding of the biodegradation process of plastics and how environmental factors influence the biodeterioration rates of these materials. Due to this, it is necessary to deepen simulation-based studies that incorporate the most relevant biological aspects in the biodegradation of resistant materials, thus allowing a feasible bioremediation process. This work shows the main mathematical models for plastic biodegradation in bioreactors and composting systems, focusing on petroleum and biological plastics.

## 2. Biochemical Features and Biodegradation Studies of Plastics

### 2.1. Biodegradation of Petroleum-Based Plastics

#### 2.1.1. Polyethylene Terephthalate (PET)

PET is the most extensively used synthetic polyester compound and is a thermoplastic of high molecular weight [15]. In the last decade, several microbial polyester hydrolases have been studied, exhibiting significant hydrolytic capacities to degrade PET [16]. Among the reported microbial polyester hydrolases, cutinases and cutinase homologues are most widely reported as capable of cutin hydrolysis [17]. Depending upon the thermal stability factor of PET, its degradation generally occurs at ∼55 °C. Thermophilic bacterial enzymes thus play a crucial role in this biodegradation process [15,18].

The more recently discovered Gram-negative bacterium *Ideonella sakaiensis* was isolated from a PET bottle-recycling factory in Japan. In contrast to all other PET degraders, *I. sakaiensis* is able to use PET as its sole source of carbon and energy by converting PET into its monomers, terephthalic acid (TPA) and ethylene glycol (EG). The two enzymes required for the conversion of PET and the intermediate mono(2-hydroxyethyl) TPA (MHET) have been identified and characterized in the literature [19]. The cutinase-like PET hydrolase, designated PETase (EC 3.1.1.101.), catalyzes the degradation of PET to bis(2-hydroxyethyl) TPA (BHET), MHET, and TPA. MHET is then metabolized by a unique hydrolase named MHETase (EC 3.1.1.102) to yield TPA, which can be used by the central metabolism via the TPA degradation pathway.

PET hydrolases are oftentimes derived from plant-cell-wall-degrading organisms, when their cutinases, lipases, or esterases have developed this side activity. Compost is generally a promising environment for novel cutin-degrading microorganisms and their extracellular enzymes. A metagenomic approach used to isolate novel PET-degrading enzymes, using a leaf-branch compost gene library, resulted in the identification and characterization of the cutinase homologue, *LC-cutinase* (LCC), with the highest sequence identities belonging to the lipase and cutinase of *Thermomonospora curvata* (*T. curvata*) and *Thermobifida fusca* (*T. fusca*), respectively [20]. The degradation rate of LCC for PET films is reported to be 200- to 900-fold higher than for previously investigated cutinases from the genera *Thermobifida* and *Fusarium*.

#### 2.1.2. Polyethylene (PE)

PE is the most abundant and commonly used fossil-based plastic, with an annual global production of 140 million tons. Its main application lies in packaging and other products with a short service time, which has led to the rapidly growing amounts of highly resistant PE waste. The recalcitrance of PE materials to biodegradation is due to the extremely stable covalent C–C and C–H bonds, as well as the absence of reactive functional groups (Figure 1). Moreover, the high molecular weight of PE, on top of its hydrophobic nature, hinders biological degradation, since extracellular enzymes capable of oxidizing and depolymerizing long carbon chains are required to break down the polymer [21]. However, little is known about the metabolic pathways, mechanisms, and enzymes involved.

The biological activity of bacteria after PE films are subjected to abiotic oxidation was observed early on in *Nocardia asteroids* and *Rhodococcus rhodochrous* [22]. *R. rhodochrous* is able to survive for 180 days on PE films pre-treated using abiotic oxidation to reduce the molecular weight of the polymer and introduce polar groups to increase hydrophilicity [23]. Strains of *Rhodococcus ruber* (*R. ruber*) and the thermophilic bacterium *Brevibacillus borstelensis* have even been shown to degrade non-pre-treated, low-density PE and use the degradation products as their sole source of carbon and energy [24,25]. For *R. ruber*, which forms a massive biofilm on the surface of the PE polymer, it has been shown that an extracellular copper-dependent laccase is mainly involved in polymer degradation. Strains of *Pseudomonas aeruginosa* are able to form biofilms on PE films pre-treated by abiotic oxidation [26,27]. Moreover, untreated low-density PE is degraded by species of *Pseudomonas* (*P. aeruginosa*, *P. putida*, and *P. syringae*), with the most efficient *P. aeruginosa* strain, PAO1, causing up to 20% weight loss of the PE material within 120 days [28]. Among the genus *Bacillus*, the first strains identified as low-density, PE-degrading microbes were *B. circulans*, *B. brevis*, and *B. sphaericus*. The strains were isolated in Japan from soil samples surrounding buried PE films and enriched using pre-treated PE powder as the sole source of carbon and energy [29]. Not only was *B. cereus* shown to degrade pre-treated PE, the enzymes responsible were identified as laccase and manganese peroxidase [30]. More recently, *B. subtilis* has been shown to degrade PE films using a biosurfactant, surfactin. Biosurfactins are amphiphilic molecules with both hydrophobic and hydrophilic domains that increase the surface area of hydrophobic water-insoluble substances such as PE [31]. Reports about the degradation of untreated PE are very limited and only describe a few bacteria from the genera *Comamonas*, *Delftia*, and *Stenotrophomonas* as being capable of breaking down high-molecular-weight PE [32].

Among fungi, PE modification or degradation has been demonstrated for *Penicillium simplicissimum* and *Cladosporium cladosporioides* [22,33], while PE microplastic is degraded by the marine fungus *Zalerion maritimum* [34].

#### 2.1.3. Polypropylene (PP)

In contrast to that of PE or PET, the microbial degradation of PP is not well investigated and only a few reports describe the degradation of pre-treated PP films by soil consortia, bacterial communities (*Pseudomonas* and *Vibrio* species), and fungal species (*A. niger*) [35,36]. Saturated polyolefins have a broad range of applications, as the versatility of these polymers arises from their cheap petrochemical feedstock origin and efficient catalytic polymerization process, and PP is one of the most widely used linear hydrocarbon polymers [37]. One of the first reports of the bacterial biodegradation of PP was in a study of cultures of the genera *Alcaligenes*, *Xanthomonas*, *Pseudomonas*, and *Vibrio*, which were found to be the predominant microaerophilic bacterial community responsible for PP degradation in a mineral medium supplemented with sodium lactate and glucose after a five-month incubation period [38]. In another study, significant fungus-mediated PP biodegradation was observed for blended and pre-treated PP material. Cultures of *Phanerochaete chrysosporium* and *Engyodontium album* allowed 18.8% and 9.42% gravimetric weight loss and 79% and 57% thermogravimetric weight loss, respectively, for UV-pre-treated, pro-oxidant blended PP over one year [39]. These results indicate that blending and pre-treatment can be successful strategies for proper bioremediation. The suitability of fungus-mediated biodegradation was proved by another study, where the endophytic fungus *Lasiodiplodia theobromae* from the plant species *Psychotria flavida* produced laccase and grew abundantly over the hydrophobic γ-irradiated PP surface over 90 days of incubation [40]. Recent work on PP biodeterioration was performed using bacteria from the genera *Bacillus* and *Rhodococcus*, which were isolated from mangrove sediment [41]. Both bacterial strains were able to utilize PP microplastics as a carbon source by means of reducing the polymer mass: 6.4% weight loss of PP was achieved by *Rhodococcus* sp. while 4.0% weight loss was reached by *Bacillus* sp. after 40 days of incubation. PP biodegradation was further confirmed using Fourier-transform infrared spectroscopy (FTIR) and Scanning electron microscopy (SEM) analyses that showed structural and morphological changes in the PP microplastics which had had bacterial treatment [41].

#### 2.1.4. Polyvinyl Chloride (PVC)

PVC is synthesized from the hydrolysis of polyvinyl acetate, and, in general, it has a low molecular weight [42]. Due to the wide range of applications of PVC in rigid or plasticized form, its production reached 5 million tons in 2018 in Europe alone [43]. Despite the extensive use of this polymer, there are only a few reports about its biodegradability. Among the PVC-degrading microorganisms, white rot fungi, *Pseudomonas* sp. and *Bacillus* sp. can be found. *P. citronellolis* and *B. flexus* were able to form a biofilm on the surface of PVC films after 45 days of incubation, achieving a 10% reduction of the molecular weight of the polymer and approximately 19% weight loss [44]. The marine bacterial strain, AIIW2, which is similar to *Bacillus* spp., was studied in terms of adhesion, degradation, and destabilization of PVC, using different techniques including SEM and FTIR analysis. It was found that the strain AIIW2 degraded PVC up to 0.26% after 90 days of cultivation [45]. Fungal strains such as *P. chrysosporium*, *Polyporus versicolor*, and four *Pleurotus* species were cultivated with PVC films and the C–H, C–Cl, and C=O bonds were analyzed as well as the impact of oxygen during cultivation. A clear decrease in the percentage of C–H bonds and increase of C–Cl and C=O was observed, influenced by the presence or absence of oxygen [46]. In other work using fungal species, the strains *P. chrysosporium PV1*, *Lentinus tigrinus PV2*, *A. niger PV3*, and *Aspergillus sydowii PV4* were isolated from a 10-month soil burial experiment in the presence of PVC films. Specifically, the fungal strain *P. chrysosporium PV1* was the most effective in terms of the reduction of the PVC film, evidenced through gel permeation chromatography (GPC), FTIR, and nuclear magnetic resonance (NMR) techniques [47]. The biodegradation yield of plasticized PVC composites, such as PVC-P and PVC-P/cellulose, is low, as shown by Kaczmarek and Bajer (2007) [48]. This might be explained by the partial cross-linking taking place during the extrusion process, making the access of aqueous enzymes to the polymers highly unlikely. Components of the PVC, such as the plasticizers, have been also studied in terms of degradation. For instance, in the work of Nakamiya et al. (2005) [49] four bacterial species were found to use the PVC plasticizers bis (2-ethylhexyl phatlate) (DEHP), where *Mycobacterium* sp. was more efficient in using this compound as its sole carbon source [49]. Recently, Giacomucci et al. (2020) [43], analyzed the biodegradation of PVC films by marine consortia using anaerobic conditions. After seven months of cultivation, a significant weight loss of up to 11.7% was reached, suggesting the degradation of the polymer chain and additives (30% w/w of the initial PVC film).

#### 2.1.5. Polystyrene (PS)

PS is considered one of the most durable synthetic polymers and is often used as styrofoam for products with a short service time. Analogous to PE degradation, the biodegradation of PS has been shown for a few bacterial strains as well as for omnivorous insect larvae. Partial biodegradation has been reported for the biofilm-forming actinomycete *R. ruber*, which is also able to degrade PE, as mentioned above [50]. Moreover, strains of *Pseudomonas* sp. and *Bacillus* sp. were found to biodegrade high-impact PS films, causing 23% weight loss within 30 days. Degradation was additionally verified by biofilm formation and the detection of structural changes and degradation products as well as FTIR analysis [51]. The fungal species *Cephalosporium* and *Mucor* were studied for the biodegradation of PS strips as their sole carbon source. A PS weight loss of 1.8–2.2% was reported, which was confirmed by SEM, FTIR, and TGA analysis. GC-MS analysis identified a list of the most important compounds that could be used as degradation products after the PS degradation assay, such as pyridine, chlorobenzene, 1,3,5 cycloheptatriene, and 2,4-diphenyl-4-methyl-2-pentene, among others [52].

Yang et al. (2015) [53], who also investigated PE-degrading larvae, showed that mealworms (the larvae of *Tenebrio molitor* Linnaeus) are able to eat and depolymerize the long-chain PS molecules of styrofoam by detecting degradation intermediates in the larval gut. That study confirmed the conversion of 48% of the PS carbon to CO${}_{2}$ and investigated the involvement of the gut microbiome in the degradation process. The PS-degrading strain, *Exiguobacterium* sp. YT2, was isolated, and PS depolymerization was confirmed by biofilm formation, bacterial growth, and, most importantly, changes in the polymer’s surface topology, a decrease in hydrophobicity, and the release of degradation intermediates [54]. Factors influencing PS degradation by yellow mealworms were shown to include: (i) the type of PS product, with less dense materials degrading faster; (ii) temperature; and (iii) supplementation of the diet, allowing for the selective breeding of a second generation of PS-degrading larvae [55]. Apparently, mealworms can be considered as a type of biorefinery, facilitating a process similar to the degradation of cellulose by microorganisms in ruminating mammals or of wood in termites, for the mutual benefit of the metabolism of microbial consortia and hosts, as pointed out by Yang et al. (2015a [54]; 2015b [53]). The physicochemical pre-treatment of the polymer by chewing and ingestion, followed by the biodegradation process inside the microbiome bioreactor, is probably a critical combination for efficient PS degradation.

#### 2.1.6. Polyurethane (PUR)

Polyester PUR coatings are widely used to help protect underlying structural surfaces but are susceptible to biological degradation. PUR-degrading bacteria have been found within the genus Pseudomonas (*P. chlororaphis*, *P. aeruginosa*, *P. fluorescens*, and *P. protegens*), and several of the enzymes involved have been purified and characterized, including polyurethane esterases A (PueA) and B (PueB) from *P. chlororaphis* and polyurethane lipase (PulA) from *P. fluorescens* [56,57,58,59]. These polyurethanes can be classified as extracellular lipases and esterases, and, in the case of *P. protegens*, it has been shown that expression of the lipases PueA and PueB is controlled by glucose carbon-catabolite repression [60]. The fungus *Aspergillus tubingensis*, isolated from the soil of a general city waste disposal site in Pakistan, degraded polyester PUR by colonizing the material using its mycelium, thereby causing surface degradation. After the growth of the mycelia, different types of enzymes are secreted [61]. Several studies have characterized PU-degrading enzymes as being able to hydrolyze the polymer [56,57,62,63,64]. Esterase and urethane hydrolase have been isolated from the fungi *A. terreus* and *Chaetomium globosum* [61], and extracellular membrane-bound lipase has been shown to be responsible for the hydrolysis of the urethane bond [65].

#### 2.1.7. Polyisoprene (PI)

Polyisoprene (PI) is a collective name for polymers that are produced by the polymerisation of isoprene. Poly(*cis*-1,4-isoprene), which is also called isoprene rubber, is the main component of natural rubber (NR). Several bacterial and fungal strains have shown the capability to degrade PI, with the genera *Gordonia*, *Streptomyces*, *Rhodococcus*, and *Xanthomonas* being some of the most studied [6]. The first oxidative attack of PI is catalyzed by rubber oxygenases, known as latex-clearing protein (Lcp), rubber oxygenase A (RoxA), and rubber oxygenase B (RoxB) [66], which incorporate molecular oxygen to cleave the double bond of PI, thus reducing the length of the polymer and forming oligoisoprenoid molecules as degradation products. The metabolism of PI in the *Gordonia polyisoprenivorans* strain VH2 has been studied, and the proposed mechanism indicates that oligoisoprenoids are transported into the cell and metabolized by β-oxidation, while acetyl-CoA and propionyl-CoA are incorporated into the central metabolism [67]. This was confirmed by the growth of *G. polyisoprenivorans* VH2 when PI was the only carbon source in the cultivation medium [68]. The main challenge is to degrade vulcanized rubber materials due to the large amounts of additives that are incorporated and the cross-linked structure, which makes it highly resistant to biodegradation [6].

For petroleum-based plastics, the use of pre-treatment by abiotic factors has been reported to either improve the biodegradation rates or, in some cases, be indispensable to biodegradation. Especially for plastics containing C–C bonds in their structure, physicochemical pre-treatments, such as UV radiation in combination with heat or chemicals [1,69], promote hydrolysis processes, making degradation by micro-organisms and their enzymatic action possible.

### 2.2. Biodegradation of Biological-Based Plastics

The production of bioplastics has increased dramatically over the last 30 years; however, it remains a small percentage of all commercially available plastics. This is mainly due to the high production costs and the occasional technical validation problems. Bioplastics made from waste or industrial by-products have become very attractive since they are a sustainable and non-polluting alternative. However, not all of these bio-based bioplastics are biodegradable [70]. Their biodegradability is determined by the route and the rate of degradation [71].

The biodegradation of the most important bio-based plastics on the market was analyzed by dividing them into three subgroups: plastics based on bio-based monomers (PLA), plastics synthesized by microorganisms (PHAs), and plastics based on renewable resources (starch and cellulose).

#### 2.2.1. Polylactic Acid (PLA)

Poly(lactic acid) is a thermoplastic synthetic polymer of the family of alpha hydroxy acids or aliphatic polyesters derived from 100% renewable raw materials, which are produced from lactic acid [72]. Although PLA can be synthesized from renewable (fermentation process) and non-renewable resources (chemical process), the former is preferred due to its low environmental impact, lower stress on fossil fuels, and greater optical clarity of the final product. Being transparent, PLA has gained wide acceptance in its use in the manufacture of packaging films, containers, and stationary products [70]. The biomedical and agricultural sectors find these polymers useful due to their biocompatibility and low carbon footprint after use [73]. It has been demonstrated that PLA bioplastic in pure form [74,75] and with additives, such as starch, softwood [74], and sisal bras [76], will experience more than 50% degradation of its biomass within three months when composted in different environmental soil samples and soil stimulants. Composting natural polymers does not affect the diversity or total biomass of live bacteria and fungi per gram of soil sample, thus presenting PLA as a viable replacement option for non-biodegradable plastics [77].

#### 2.2.2. Polyhydroxyalkanoates (PHA)

PHAs comprise a group of naturally occurring biodegradable polyesters that are synthesized by microorganisms, including through bacterial fermentation of lipids or sugars. The market price of PHA is considered to be relatively high in comparison to the costs of conventional polymers, such as PP and PE [78]. PHA can be degraded naturally by microorganisms through enzyme-driven hydrolytic cleavage reactions, yielding water-soluble monomers and water-soluble oligomers that can be further metabolized to carbon dioxide and water under aerobic conditions and into methane under anaerobic conditions [79]. The complete biodegradation of PHA films can occur within a few months in many environments (fresh or marine water, activated sludge, and soil). However, since an active microbial environment is required for degradation, PHAs were found to be stable to humidity and exposure to air [80]. The addition of low-cost natural and inorganic fillers may allow for the production of lower cost PHA-based composites that can be applied in single-use products, especially in some industrial sectors, such as packaging and agriculture, where the biodegradability of PHA in compost, soil, and seawater represents a major advantage [81].

#### 2.2.3. Starch-Based Polymers

Starch is a naturally occurring polysaccharide that can be obtained from a wide variety of crops, such as cassava and maize. It is considered one of the most abundant renewable materials known to man. It is edible and biodegradable, which makes it an attractive material for food packaging. It can exhibit thermoplastic properties in the presence of plasticizers, such as water and glycerol, at high temperatures (90–180 °C), and under shear stress [78]. Starch-based polymers are made of starch mixed with thermoplastic polyesters to form biodegradable and compostable products. Blends of starch with aliphatic polyesters improve their processability and biodegradability, with polycaprolactone (PCL) and aliphatic polyesters being the most suitable described polyesters. These blends are used to manufacture high-quality packaging films and laminates [82]. The mechanism of biodegradability is influenced by environmental parameters, such as the microbiome present, as well as by external factors, such as temperature, light, oxygen, water, pressure, ozone, etc. In general, starches are degraded into glucose by microorganisms or enzymes and then metabolized into carbon dioxide and water [83].

#### 2.2.4. Cellulose-Based Polymers

Cellulose, a polysaccharide, is one of the most prevalent biopolymers used as natural alternative packaging. Cellulose is made up of -d-glucose subunits, and its polymers are obtained from plants. In its native form, cellulose has very low water solubility and is, therefore, a rather unsuitable substance for packaging material. Functionalized thermoplastics made from cellulose diacetate and triacetate exhibit higher tensile strengths and resistances to heat and water. The cellulose acetate (CA) fibers are recyclable; they are easily incinerated without leaving residues and decompose ecologically in both soil and water [70]. CA made from linen fibers and cotton lint was found to have lost 44% and 35% of its initial mass, respectively, within 14 days after incubation in compost simulated conditions [84]. In turn, cellulose-based sponge cleaners can degrade 20–50% of their initial mass in 22 weeks [85].

## 3. Main Techniques to Evaluate Solid Waste Biodegradation

In order to analyze the degradation process of solid pollutants such as plastics, it is necessary to establish the methodology to demonstrate such degradation. The main techniques used are described below and can be shown as a single technique or in combination (see Table 1).

### 3.1. CO${}_{2}$ Measurement

This methodology provides a percentage value of material biodegradation by measuring the organic carbon transformed into gaseous CO${}_{2}$ during the aerobic degradation process [86]. CO${}_{2}$ measurement can be performed with different equipment such as cumulative measurement respirometry (CMR) [87], gravimetric measurement respirometry (GMR) [88], and direct measurement respirometry (DMR) [89]; the latter is equipped with a non-dispersive infrared sensor. Another way to quantify CO${}_{2}$ is by means of the biological oxygen demand (BOD), which involves measuring the decrease in oxygen pressure and the simultaneous absorption of the produced CO${}_{2}$ (Oxitop) [90].

### 3.2. Mass Loss

Mass loss is considered by many authors as an index of biodegradation, indicating the decrease in molecular weight or assessing the degree of disintegration [91]. For the measurement of molecular weight, GPC (SEC) is used, where molecules in solution are separated according to their size or molecular weight [92]. Another method used is TGA, which is thermal analysis, where the mass of the sample is measured over time as the temperature changes [89]. Mass-loss measurement is frequently used, which involves measuring the degree of disintegration according to standards that normalize the compostability of plastics according to the percentage of particles that are retained on a 2 mm sieve [93].

### 3.3. Spectroscopy

This technique is used to evaluate the biodegradation process through the surface changes of a material [94]. For solid materials, the technique of attenuated total reflectance coupled with Fourier-transform infrared radiation (ATR-FTIR) is very practical, providing details about the changes undergone by the main functional groups that make up the material after a degradative process [95]. For this, the absorption or transmission spectra of samples are analyzed in the wavelength of 4000–400 cm−1. X-ray-induced photoelectron spectrometry is a method that involves measuring the spectra of photoelectrons induced by X-ray photons [21]. Another very powerful technique is NMR, which delivers the sequence of active nuclei, usually expressed on the basis of C, H, and O [96].

### 3.4. Microscopy Analysis

Much of the research in the field of the biodegradation of solid pollutants uses visual microscopy analysis to confirm or complement the results obtained with previously analyzed methodologies [97]. Using SEM, the crack formation, surface roughness, and corrosive degradation of samples are analyzed [98]. Atomic force microscopy (AFM) allows for the characterization of the nanostructure of the crystal surface in solution [21]. In turn, many studies use photographs to report the generality of the color, size, roughness, and characteristics of plastic materials during the degradation period [99].

## 4. Biodegradation of Polymers under Controlled Conditions

### 4.1. Biodegradation in Simulated Composting Systems

Composting is an aerobic biological process that, under controlled aeration, humidity, and temperature conditions, transforms degradable organic waste into a stable and sanitized material called compost. In Table 2, we present the different studies that have been published about the biodegradation of plastic materials in aerobic composting systems with their respective work methodologies (according to Ruggero et al., 2019 [100]).

In Sarasa et al. (2009) [75], the degree of biodegradation of different pieces made of a biodegradable material (PLA with and without corn in its composition) was studied. The parts made with PLA and PLA-corn were subjected to aerobic degradation at a constant temperature of 58 ± 2 °C for 90 days, following the EN 14806 and ISO 20200: 2004 Standards. The PLA and PLA pieces containing a foaming agent were found to have an average degree of biodegradation of 63.6%. With respect to the PLA-corn pieces, an average degree of biodegradation of 79.7% was obtained. Pradhan et al. (2010) [92] evaluated the degree of degradation of PLA and injection-molded compounds of PLA-wheat straw (70:30) and PLA-soybean straw (70:30). The experiment was performed on a laboratory-scale simulated composting system (per ASTM D 5338). The results of the study revealed the suitability of the test protocol and the validity of the test system and defined the compostability of the PLA compounds with unmodified natural substrates. In Arrieta et al. (2014) [98], the biodegradation of poly(lactic acid)-poly(hydroxybutyrate) (PLA–PHB) under composting conditions was studied. Disintegration levels were evaluated by monitoring their weight loss at different times: 0, 7, 14, 21, and 28 days. The ability of PHB to act as a nucleating agent in PLA-PHB mixtures slowed down the biodegradation of PLA, whereas plasticizers accelerated it. The relationship between the mesolactide and lactide forms of PLA was calculated with a pyrolysis-gas chromatography–mass spectrometry (Py-GC/MS) device, revealing that the mesolactide form increased during composting. Luzi et al. (2016) [104] investigated the biodegration under composting conditions of different bionanocompounds based on PLA and PLA-PBS. The presence of cellulose nanocrystals, both unmodified (CNC) and with modified surfactant (s-CNC), and the addition of PBS to the PLA matrix led to an improvement in barrier properties. The biodegradation under composting conditions revealed that the presence of surfactants facilitates the biodegradation, while the presence of PBS reduces the biodegradation values. In any case, all the bionanocomposites were biodegraded in less than 17 days. In the work of Fortunati et al. (2014) [93], a study was carried out on the biodegradation of bio-nanocomposite PLA films modified with limonene (Lim) as a plasticizer and reinforced with cellulose nanocrystals (CNC) under simulated composting conditions. The study involved a series of phases, starting from the extraction of CNC from phormium leaves, then going on to the optimization of the plasticizer content, and finally reaching the production of the ternary biocomposites PLA-Lim-CNC. Finally, it was shown that the presence of both a plasticizer and CNC can alter the degradation rate of formulations developed based on PLA.

In Mohee et al. (2008) [96], a study was carried out on two types of plastic materials, Mater-Bi Novamont (MB) and Environmental Product Inc. (EPI), to evaluate their biodegradability under aerobic and anaerobic conditions. Cellulose filter papers (CFP) were used as a positive control for both mediums. In the aerobic test, the mass of MB decreased by 26.9%, which implies that it did not completely biodegrade within that period of time and it required more time to fully biodegrade, and no mass decrease was observed for the EPI. In the work of Song et al. (2009) [101], the potential impacts of biodegradable packaging materials and their waste management through composting were analyzed. Key questions about the benefits these materials have relative to their conventional petrochemical-based counterparts were presented. Examples of new research on biodegradability in simulated home composting systems were given. It was concluded that biodegradable packaging materials are more suitable for single-use disposable applications where post-consumer waste can be converted into local compost. In Lavagnolo et al. (2017) [105], the fate of bioplastics during the composting process was investigated, in order to follow their biodegradation and disintegration until they were released into the environment. Analysis carried out on a laboratory scale showed that bioplastics have different potentials depending on the polymers that compose them [106], or according to the temperature of the composting process [96,103].

In the work of Gómez and Michel (2013) [102], the rate and degree of mineralization of a wide range of commercially available alternative plastic materials were determined during composting, anaerobic digestion, and soil incubation. The reactors were aerated at 100 ± 1 mL/min to maintain aerobic conditions. The results showed that, during a 660 day soil incubation, substantial mineralization was observed for PHA plastics, starch-based plastics, and materials made from compost. However, only one PHA-based plastic showed a similar biodegradation rate compared to the positive control (cellulose). In Javierre et al. (2015) [103], the influence of painting starch-based plastic bags was studied. The degree of disintegration (D%) of a 100% biocompostable plastic made from potato starch was calculated for both painted and unpainted samples. The laboratory-scale composting process was carried out following the UNE-EN standards. It was concluded that the paint had a negative influence on the biodegradation process, decreasing the degree of disintegration by 4.48%. The negative effect of the paint is due to the barrier effect of the paint that prevents microorganisms from converting the organic components of the biopolymer into water, CO${}_{2}$, and compost.

### 4.2. Biodegradation Using Stirred Bioreactors

A stirred tank bioreactor is a vessel that maintains a biologically active environment, in which a chemical process is carried out that involves organisms or biochemically active substances derived from said organisms. For its operation, environmental conditions such as gas flow (for example, oxygen, nitrogen, carbon dioxide, etc.), temperature, pH, dissolved oxygen, and stirring or circulation speed must be guaranteed. Unlike composting systems, when using bioreactors, the levels of control of the operating parameters are more precise, maintaining greater process stability. Studies focused on the BD of plastics on stirred bioreactors are shown in Table 3 with their respective work methodologies (according to Ruggero et al., 2019 [100]).

The analysis of previously published studied regarding the biodegradation of PLA in stirred bioreactors are shown below. In MassardierNageotte et al. (2006) [90], an Oxitop respirometer was used for the aerobic testing of the biodegradation of different plastic materials: i.e., MB, PCL, PE, PLA. After 28 days, it was shown that the degradation of the different polymers depended on the material and on the test conditions used. The degradation was better under aerobic conditions, in particular for Mater-Bi and polycaprolactone. Nevertheless, the study found that the amorphous parts of the polymer was more easily colonized by the micro-organisms, but, after 28 days, they did not seem to cleave macromolecules inside the material. As expected, bacteria attacked the surface of the polymer and seem to consume the macromolecules one after another. In Kale et al., 2007 [88], two simulated composting methods were used to assess the biodegradability of PLA bottles: (a) a cumulative measurement respirometric system (CMR) and (b) a gravimetric measurement respirometric system (GMR). Both the CMR and GMR systems showed similar biodegradation trends for PLA bottles, and, at the end of day 58, mineralization was 84.2 ± 0.9% and 77.8 ± 10.4%, respectively. In Iovino et al. (2008) [107], the aerobic biodegradation of a PLA compound was investigated under controlled composting conditions using standard testing methods (ISO 14855). A glass flask bioreactor containing 2 L of culture medium was used. For this, the compost and the test materials were mixed in a ratio of 6:1 (calculated on dry mass) and then placed in an oven at a constant temperature of 58 ± 2 °C maintained during the 90 days of the experiment. Finally, the glass flasks were shaken and weighed weekly to ensure adequate aeration and mixing of the bio-waste. The degradation results were confirmed by SEM analyses of the aged compost samples. In Petinakis et al. (2010) [89], the effect of hydrophilic fillers (starch and wood flour) on the BD of PLA-based materials was investigated by composting under controlled conditions according to ISO 14855. Three replicates of each sample type were prepared and BD tests were performed in a National Association of Testing Authorities (NATA) certified respirometric unit for 85 days. During the course of this test, the CO${}_{2}$ released from each compost bin was measured at intermediate time intervals using an infrared analyzer and the percentage degree of BD was calculated. Biodegradation rates of PLA/starch blends and PLA compounds/wood flour were found to be lower than pure cellulose but higher than pure PLA. In Leejarkpai et al. (2011) [109], the kinetics of the evolution of C–CO${}_{2}$ during the biodegradation of plastic materials (PE, PE/starch, MCE and PLA) was evaluated. The experimental work was carried out in two-liter glass reactors. The work showed that MCE and PLA produced high amounts of evolution of C–CO${}_{2}$, which gave easily hydrolyzable carbon values of 55.49% and 40.17%, respectively, with rapid hydrolysis rates of 0.338 day${}^{-1}$ and 0.025 day${}^{-1}$, respectively. On the other hand, a lower amount of C–CO${}_{2}$ release was found in PE/starch, which had a high concentration of moderately hydrolyzable carbon of 97.74% and a moderate hydrolysis rate of 0.00098 day${}^{-1}$. The mineralization rate of PLA was 0.5 day${}^{-1}$, since a lag phase was observed at the beginning of the biodegradability test. No lag phase was observed in the biodegradability tests of PE/starch and MCE. In Cadar et al. (2012) [110], aerobic biodegradation was carried out under controlled composting conditions of PLA and commercially available synthesized co-polymers in the laboratory according to ISO 14855-1:2005. Each biodegradation study was performed in duplicate in 2 L glass flasks containing: (i) test material (compost and polymeric material), (ii) reference material (compost and microcrystalline cellulose, MCE), and (iii) a negative control (compost without polymeric material). The biodegradability of tested materials was found to be strongly dependent on the lactic acid content, ranging from 94% (method A) and 104% (method B) to 43% (method A) and 46% (method B) over the 110-days of the 50 °C composting.

Several biodegradation studies of PHA and PHB in agitated bioreactors were compiled, which are discussed below. In Weng et al. (2010) [108], the biodegradation behaviors of PHBV films (3 mol% HV) under different conditions were investigated on a pilot and laboratory scale according to ISO 16929 and ISO14855-2, respectively. For each container, 60 g of mature compost (based on dry solids) was placed in a composting bioreactor, and the water content of the compost was adjusted to approximately 110% of the water holding capacity. The PHBV film was completely disintegrated in the pilot scale composting test, while the degree of biodegradation for the laboratory scale composting test reached 81%. In Weng et al. (2011) [95], the influence of the chemical structure on the biodegradability of PHA was analyzed. Furthermore, the biodegradation behavior of PHB, PHBV (40% mol HV), PHBV (20% mol HV), PHBV (3% mol HV), and P (3HB, 4HB) (10% mol of 4HB) under controlled composting conditions was analyzed in accordance with ISO 14855-1. The test reactor contained the material for composting (2.5 L), an air supply system, and the apparatus for the determination of CO${}_{2}$ with a continuous infrared analyzer. A total of 100 g of test material was mixed with 600 g of inoculum and then placed in a composting container where it was composted under the optimal oxygen level using an air supply system. According to the authors, the oxygen concentration was not less than 6%, and the temperature was set at 58 ± 2 °C. Furthermore, the authors pointed out that the trial period should not exceed six months. It was concluded that PHAs with different chemical structures can biodegrade under controlled composting conditions. In Tabasi et al. (2015) [106], the ATR-FTIR technique was used in combination with a laboratory-scale composting setup to investigate the selective composting of two-phase biodegradable mixtures based on PLA or PHB. The ATR-FTIR analysis showed that the mixtures were rich in polybutylene adipate terephthalate (PBAT) content, indicating the selective degradation of PLA or PHB components in the mixtures. The investigation of the mechanical properties of the mixtures demonstrated a gradual loss of Young’s modulus due to growing defects caused by either hydrolysis or corrosive enzymatic degradation, which further caused a decrease in molecular weight.

In the work of Du et al. (2008) [99] the degradability of thermoplastic starch (TPS) and thermoplastic dialdehyde starch (TPDAS) was investigated under controlled composting conditions according to ISO 14855. All materials were ground into a fine powder in advance. Two blank reactors and two reference reactors were included in this biodegradation testing system. To determine the CO${}_{2}$ released, they used a test system consisting of three parts: (i) a pressurized air control system, which was a pre-treatment used to remove CO${}_{2}$ from the compressed air as a carrier gas to control the aeration rate and to prepare saturated aqueous vapor; (ii) a composting system containing a mixture of test material and inoculum; and (iii) a CO${}_{2}$ cheat system. The TPS and TPDAS biodegradation processes exhibited three phases or stages with different degradation rates. In the first phase, biodegradation was slow, it was accelerated in the second phase, and stabilized in the third phase. In Pischedda et al. (2019) [111], the evaluation of the intrinsic biodegradability of plastic materials was carried out under optimized environmental conditions so as not to limit growth and microbial activity and to follow the biodegradation process until its completion. It was concluded that temperature affects biodegradation rates, according to the Arrhenius equation. The observation that the apparent activation energy of the biodegradation reaction does not vary with temperature in the tested temperature range indicates a persistence in the metabolic activities of the mesophilic microbial communities involved. Finally, it was observed that the compositions or chemical structures of plastic materials are essential, and one of the most important influencing factors for biodegradation studies was aeration.

## 5. The Most Common Mathematical Models for Plastic Biodegradation

In the literature, there are many studies about the kinetic modeling (KM) of the reaction for the degradation of plastic materials (PMs). Each of the mathematical models (MMs) for polymer biodegradation that have been found in this literature review over the last 20 years are organized in Table 4.

Jhonson et al. (2010) [112] studied the influence of different degrees of carbon and nitrogen limitations on the performance of polymer biodegradation using a sequencing batch reactor (SBR) fed with acetate used to enrich PHA-storing bacteria. It was found that the microbial reaction rates in the SBR showed a change in the limiting substrate. Komilis et al. (2006) [113] considered a kinetic model of composting that described the C mineralization of various organic materials, and the study was carried out considering only the growth phase and stationary phase. Subsequently, Leejarkpai et al. (2011) [109] proposed the same model as Komilis et al., plus the lag phase. It was assumed that the first stage of external degradation consisted of rapidly, moderately, and slowly hydrolysable solid carbon. Cell degradation was assumed to be the mineralization of intermediate solid carbon to carbon dioxide (CO${}_{2}$). According to ISO 14855, the theoretical amount of CO${}_{2}$ (${\mathrm{ThCO}}_{2}$), in grams per reactor, is calculated using the following equation:

(1) $${\mathrm{ThCO}}_{2}=M_{TOT}\times C_{TOT}\times \frac{44}{12},$$

where ${\mathrm{ThCO}}_{2}$ is the theoretical amount of CO${}_{2}$ that can be produced by the plastic sample, in grams per reactor; $M_{TOT}$ is the total dry solid in grams in the plastic sample added to the compost reactor at the start of the test; $C_{TOT}$ is the ratio of total organic carbon to total dry solid in the plastic sample, in grams per gram; and 44 and 12 are the molecular mass of CO${}_{2}$ and the atomic mass of carbon, respectively. The total amount of CO${}_{2}$ evolution was calculated by reference to the blank control reactor. A biodegradation curve was obtained, which represented graphically the CO${}_{2}$ released (%) versus the exposure time. The biodegradation of the plastic sample was calculated as the percentage of carbon mineralized as CO${}_{2}$ according to the following equation:

(2) $$\left(\%\right)Biodegradation=\frac{{\left({\mathrm{CO}}_{2}\right)}_{T}-{\left({\mathrm{CO}}_{2}\right)}_{B}}{{\mathrm{ThCO}}_{2}}\times 100,$$

where ${\left(CO_{2}\right)}_{T}$ and ${\left(CO_{2}\right)}_{B}$ are the cumulative amount of CO${}_{2}$ released in the reactor with sample and in the blank reactor, respectively. Equations (1) and (2) are widely used by researchers to measure the degradation percentages of plastic samples under aerobic composting conditions.

**Table 4:** Main mathematical models describing the biodegradation of petroleum- and bio-based plastics.

Reference	Plastic	Model	Discussion
[112] Jhonson et al., 2010	PHB	${\tilde{q}}_{PHB}^{fam}=k\cdot {\tilde{f}}_{PHB}{\left(t\right)}^{2/3}$	This paper studied the influence of C/N in the production of PHB.
[109] Leejarkpai et al., 2011	MCE,	$\frac{dC_{r}}{dt}=-k_{hr}\cdot C_{r}$,	This paper studied the KM of the evolution of CO${}_{2}$ during the BD of PMs.
	PLA,	$\frac{dC_{m}}{dt}=-k_{hm}\cdot C_{m}$,	
	PE,	$\frac{dC_{s}}{dt}=-k_{hs}\cdot C_{s}$,	
	PE/starch	$\frac{dC_{aq}}{dt}=k_{hr}C_{r}+k_{hm}C_{m}$	
		$+k_{hs}C_{s}-k_{aq}C_{aq}$	
		$\frac{dC_{T}}{dt}=k_{aq}\cdot C_{aq}$	
[114] Farzi et al., 2019	PET,	$\frac{dC_{A}}{dt}=-r_{A}$	The BD of PET by the *Streptomyces*
		$-r_{A}=\frac{kC_{A}^{2}}{1+k_{1}+k_{2}C_{A}^{2}}$	species was evaluated using Arrhenius and M–M models.
		$-r_{A}=\frac{kC_{A}}{1+k_{1}+k_{2}C_{A}^{2}}$	
[111] Pischedda et al., 2019	Mater-Bi	$k=Ae^{-\frac{E_{a}}{RT}}$	This study concluded that the rate of BD
	MCE		in the soil is affected by temperature.
[12] Chamas et al., 2020	PP, PVC,	$-\frac{dm}{dt}=k_{d}\rho \mathrm{SA}$	Plastic degradation rates and pathways under various conditions were studied.
	PET, PS,		
	H and LDPE		
[115] Sánchez et al., 2021	PS, EPDM	$\frac{dx}{dt}=k_{T}\left(1-x\right)$	This study concluded that selecting the wrong KM affects BD predictions.
	PET, PLA	$=Ae^{-\frac{E}{RT}}\left(1-x\right)$	
[116] Perejon et al., 2021	PI	$\frac{d\alpha }{dt}=Ae^{-\frac{E}{RT}}f\left(\alpha \right)$	This study showed that the thermal decomposition of natural rubber is predicted.
[117] Rossetti et al., 2021	Commercial	$\ln(m\left(t\right)/m^{\circ })=kt$	BD methods of different plastic materials were compared.
	plastics	$\frac{1}{v}=\frac{1}{v_{max}}+\frac{K_{M}}{v_{max}\left[S\right]}$	
	Cellulosa	$\frac{dn}{dt}=k\;n^{n}$	
[11] Ruggero et al., 2021	MB, PBAT,	$PA\left(\%\right)=\int_{T_{0}}^{T_{inf}}\frac{dw}{dT}dT$	Bioplastic degradation during the thermophilic phase was studied.
	PLA, LDPE	$WL\left(\%\right)=100-\frac{PA}{PA_{0}}\cdot 100$	

Aerobic biodegradation under controlled composting conditions of PE (0.6%), PE/starch blend (PE/almidón, 12%), PLA (86%), and microcrystalline cellulose (MCE, 94%) was performed according to ISO 14855-1, 2004. SEM characterization of the degraded material was used to confirm the results of the biodegradability tests. This model has been considered as a base mathematical kinetic model by many researchers in their studies on the biodegradation of plastic materials under controlled composting conditions.

One of the important factors to consider in the biodegradation process of plastics is temperature. To involve this parameter, many authors have incorporated the Arrhenius equation into kinetic models, which is given by the following expression:

(3) $$k\left[T\right]=Ae^{-\frac{E_{a}}{RT}},$$

where *k* is the reaction rate, *A* is the pre-exponential factor, $E_{a}$ is the activation energy of the reaction, *R* is the gas constant, and *T* is the temperature in degrees Kelvin. The Arrhenius Equation (3) makes it possible to verify the dependence of the rate constant of a chemical reaction on temperature. In a study by Chamas et al. (2020) [12], a general description of the degradation rates (by mass loss) of plastics in the environment is given, with a summary of the current knowledge on the degradation rates of different types of basic plastics under various environmental conditions by means of the Arrhenius equation.

In the work of Perejon et al., (2021) [116], a method is proposed to study the kinetics of complex processes composed of non-independent stages. The method consists of the simultaneous kinetic analysis of a set of experimental data recorded under linear heating rate conditions, with no prior assumptions about the kinetic patterns followed by the stages or their corresponding activation energies. This method has been tested using the kinetic analysis of the pyrolysis of NR, since the kinetics of this process are complex and depend on the temperature and heating schedule. Pyrolysis is the thermal degradation of a substance in the absence of oxygen, so these substances are decomposed by heat, without combustion reactions taking place. Pyrolysis is one of the important solutions for recycling rubber waste. The natural rubber pyrolysis process consists of two stages. This behavior can be explained taking into account that during the pyrolysis of NR, the depolymerization, condensation, and degradation reactions take place at the same time. It was shown that the behavior of the experimental curves of the thermal decomposition of NR can be predicted accurately with the kinetic parameters calculated by the proposed methodology. Pischedda et al. (2019) [111] studied the effects of temperature on the biodegradation of plastics in soil. In this article, the biodegradation rate of biodegradable plastics (e.g., Mater-Bi, microcrystalline cellulose) after accidental or deliberate release into the environment was estimated by means of the Arrhenius equation. This study is an initial step towards the development of a methodology to simulate field dissipation kinetics, taking into account the effects of soil temperature. Recently, in a study by Sanchez et al. (2021) [115], predictions were made for the thermal degradation of PLA, PET, PS, and ethylene propylene diene terpolymer (EPDM) polymers. It concluded that any kinetic analysis of a chain scission-driven reaction carried out assuming a first-order model involves huge deviations in predictions. Therefore, the kinetic analysis procedure must be carefully selected if any kind of extrapolation to conditions other than experimental is to be attempted.

When working with biodegradation processes, it is the enzymes of the microorganisms that catalyze the fractionation reactions corresponding to the structure of each polymer. For this reason, understanding the enzyme kinetics of depolymerization reactions is of great relevance, and the Michaelis–Menten (M–M) equation has been the most employed equation in biocatalytic processes. The M–M equation is as follows:

(4) $$v=\frac{V_{max}\left[S\right]}{K_{M}+\left[S\right]},$$

where *v* is the rate of product formation, $V_{max}$ is the maximum rate of the reaction, $K_{M}$ is the Michaelis–Menten constant, and [S] is the substrate concentration. The M–M Equation (4) describes the rate of reaction of many enzymatic reactions and indicates the number of substrate molecules that are converted to product per second. In a study conducted by Farzi et al. (2019) [114], the biodegradation of PET residues was studied using *Streotomyces* species using three laboratory-scale kinetic models. The first model is called the power law model (based on the Arrhenius equation), and the other two (based on the M–M equation) are called (i) the M–M inhibition model and (ii) the M–M activation model. Bacteria were shown to degrade PET dusts into less harmful components with low carbon content. Furthermore, it concluded that the inhibition and activation models of M–M are proposed as the best kinetic models for predicting the BD of PET powder samples. In a previous study, Rossetti et al. (2021) [117] compared the biodegradation methods of different commercial plastic materials obtained under aerobic composting conditions, following ISO 14855. From the raw data, the conversion vs. time inputs were developed using relatively simple kinetic models, such as zero-, first-, and second-order integrated kinetic equations, through the Wilkinson model or using a M–M approach. The M–M approach fails to describe all reported kinetics, as well as zero- and second-order kinetics. The BD pattern of a sample was described in detail by simple first-order kinetics. In contrast, other substrates followed a more complex pathway, involving rapid partial degradation, which subsequently slowed down. Therefore, a more conservative kinetic interpolation was needed. In addition, the different possible patterns were discussed, with guidance provided for the application of the most appropriate kinetic model.

Finally, Rugero et al. (2020 [118]; 2021 [11]) studied mass-loss-based models. Rugero et al. (2020) [118] studied the results of a laboratory-scale composting test on a Mater-Bi (MB) film were carried out, which was composed of starch, additives, and polybutylene adipate terephthalate (PBAT). The test lasted 45 days and was carried out in three replicates under different temperature and humidity conditions in order to evaluate these conditions’ influence on MB degradation under less favorable composting conditions. The results show that the biodegradation of PBAT is strongly influenced by environmental conditions (temperature and humidity). In contrast, in all three replicates, both starch and additives were completely biodegraded in the first days of the process. Rugero et al. (2021) [11] studied the effective degradation of bioplastic products under composting. The composting test was performed using MB, PBAT, and PLA of different thicknesses, taking 20 days for the thermophilic phase, followed by 40 days for the maturation phase. The most interesting observations were made for PLA, which was strongly influenced by both the thickness and the duration of the thermophilic phase, which was shorter than EN 13432 conditions.

## 6. Conclusions

The biodegradation of synthetic plastics has become one of the main environmental concerns due to the continuous accumulation of highly durable materials and the generation of micro-plastics. For the first time, a compilation of the main equations and mathematical models involved in the biodegradation processes of plastics of both petrochemical and biological origin is presented. We hope that this review will be useful for future research combining the modeling, experimentation, optimization, and application of large-scale biodegradation processes. Collecting those mathematical models currently applied by the scientific community and identifying their behaviors and trends will provide a clearer notion of the context in which the biodegradation processes of petroplastic and bioplastic materials such as those mentioned in this study could take place, as well as showing how each factor influences the biodegradation process. The mathematical modeling of the biodegradation of different plastics has shown that it is possible to simulate experimental conditions and to evaluate the effectiveness of the degradation process over time. The main influential factors, such as temperature, aeration, humidity, substrate composition, and particle size, have been identified in order to provide optimal conditions for microorganisms to grow and attack plastic surfaces. However, to fully exploit the mathematical tools in terms of simulations and predictions, more work needs to be completed to pave the way for designing efficient biological and/or enzymatic degradation processes. Different polymer blends are being developed to fulfill the current market demands, and, therefore, the theoretical prediction of the biodegradation rates of newly plastic composites can be identified as a relevant tool for the industry.

There is an important difference in the biodegradation rates when comparing synthetic petrochemical plastics or bio-based biodegradable plastics, mainly the presence or absence of reactive functional groups, the stability of the chemical structures of the different plastics, and the addition of external compounds as stabilizers that make many commercial plastics a hard and undesirable substrate for most microorganisms. It is evident that biodegradability is not a straightforward feature, since environmental conditions affect the speed and development of the degradation process. Physico-chemical pre-treatments for some petroleum-based plastics (mainly C–C plastics) are required prior to the biodegradation step. In this way, modeling of multi-step bioprocesses seems to be necessary to evaluate the biodegradation yields of the most persisting plastics.

## Figures and Tables

**Table 1:** Methodologies used to evaluate the biodegradation of plastic materials.

Group	Methodology
CO${}_{2}$ measurement	CMR: Cumulative measurement respirometry
	GMR: Gravimetric measurement respirometry
	DMR: Direct measurement respirometry
	Oxitop
Mass loss	GPC: Gel permeation chromatography
	SEC: Size exclusion chromatography
	TGA: Thermogravimetric analysis
	Experimental mass loss
	Disintegration degree
Spectroscopy	XPS: X-ray photoelectron spectroscopy
	FTIR: Fourier transformation infrared
	NMR: Nuclear magnetic resonance
	NIR: Near infrared
Microscopy analysis	SEM: Scanning electron microscopy
	AFM: Atomic force microscope
	Photographs

**Table 2:** Analysis of plastic biodegradation studies in composting systems.

Author	Plastic	Days	T (°C)	BD (%)	Methodology
[96] Mohee et al., 2008	CFP	72	30 ± 2	100	Mass loss
	MB	72	30 ± 2	26.9	SEC, DSC
	EPI	100	30 ± 2	0.02	NMR, FTIR
[75] Sarasa et al., 2009	PLA	90	58 ± 2	63.6	Mass loss
					Disint. degree
[101] Song et al., 2009	PLA	90	S.C.	5	Mass loss
	Mater-Bi	90	S.C.	5	
[92] Pradhan et al., 2010	PLA	100	58 ± 2	90	CO${}_{2}$ measurement
					SEC, GPC
[102] Gómez and Michel, 2013	PHA	660	S.C.	70	CO${}_{2}$ measurement
	Plastarch	660	S.C.	30	DMR, SEM
[98] Arrieta et al., 2014	PLA	35	58 ± 2	90	Desint. degree
	PHB	35	58 ± 2	90	SEM, TGA, FTIR
[93] Fortunati et al., 2014	PLA	14	58 ± 2	90	Disint. degree
[103] Javierre et al., 2015	Starch-based	90	58 ± 2	85	Disint. Degree
[104] Luzi et al., 2016	PLA	90	58 ± 2	90	Disint. degree, SEM
[105] Lavagnolo et al., 2017	Mater-Bi	55	S.C.	80	Mass loss, FTIR

S.C.: Simulated Composting in two temperature stages (thermophilic phase 58–65 °C and mesophilic phase 25–40 °C).

**Table 3:** Analysis of plastic biodegradation studies in stirred bioreactors.

Author	Plastic	Days	T (°C)	BD (%)	Methodology
[90] Massardier-Nageotte et al., 2006	Mater-Bi	28	30 ± 2	42.8	Oxitop
	PCL	28	30 ± 2	34.8	Mass loss
	PE	28	30 ± 2	4.1	FTIR, NMR
	PLA	28	30 ± 2	3.7	SEC, DSC
[88] Kale et al., 2007	PLA	30	65	95	GMR, GPC
[99] Du et al., 2008	Starch-based	56	58 ± 2	73.11	CMR, Photographs
[107] Iovino et al., 2008	Starch-based	90	58 ± 2	87	DSC, CMR
	PLA	90	58 ± 2	55	SEM
[89] Petinakis et al., 2010	PLA	80	58 ± 2	60	DMR, TGA, SEM
[108] Weng et al., 2010	PHB	39	58 ± 2	81	SEM, FTIR
[109] Leejarkpai et al., 2011	MCE	90	58 ± 2	94.34	CO${}_{2}$ measurement
	PLA	45	58 ± 2	85.75	SEM
	PE	90	58 ± 2	0.56	
	PE/starch	90	58 ± 2	11.50	
[95] Weng et al., 2011	PHB	110	S.C.	79.7	FTIR, SEM
					Desint. degree
[110] Cadar et al., 2012	PLA	110	50	70	Desint. degree, CMR
[106] Tabasi et al., 2015	PHB	30	55	70	GMR, FTIR, SEM
	PLA	30	55	70	
[87] Balaguer et al., 2016	PLA	130	58 ± 2	90	CO${}_{2}$ measurement
					CMR, TGA
					Desint. degree
[111] Pischedda et al., 2019	Mater-Bi	28	28	56.4	CO${}_{2}$ measurement
	MCE	28	28	44.4	

S.C.: Simulated Composting in two temperature stages (thermophilic phase 58–65 °C and mesophilic phase 25–40 °C).
